# QuickStats

**Published:** 2015-07-24

**Authors:** FROM THE NATIONAL CENTER FOR HEALTH STATISTICS

**Figure:**
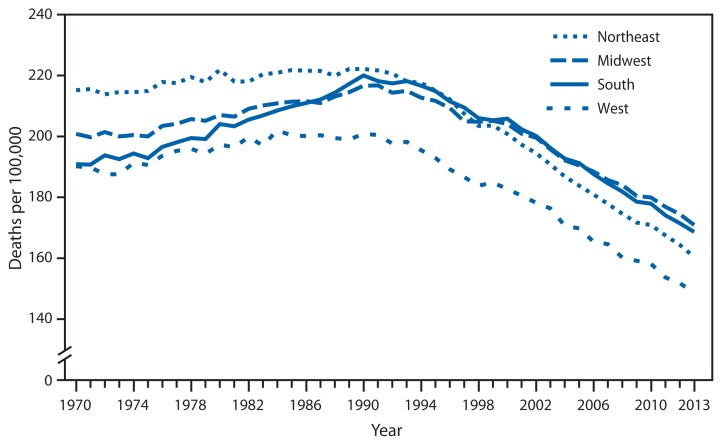
Age-Adjusted Death Rates* from Cancer,^†^ by U.S. Census Region^§^ and Year — United States, 1970–2013 * Per 100,000 standard 2000 population. ^†^Cancer deaths are identified using underlying cause of death with codes 140–209 (1970–1978), 140–208 (1979–1998) and C00–C97 (1999–2013) in the *International Classification of Diseases, Eighth, Ninth, and Tenth Revision*. ^§^ *Northeast*: Connecticut, Maine, Massachusetts, New Hampshire, Rhode Island, New Jersey, New York, Pennsylvania, and Vermont; *Midwest*: Illinois, Indiana, Iowa, Kansas, Michigan, Minnesota, Missouri, Nebraska, North Dakota, Ohio, South Dakota, and Wisconsin; *South*: Alabama, Arkansas, Delaware, Florida, Georgia, Kentucky, Louisiana, Mississippi, Maryland, North Carolina, Oklahoma, South Carolina, Virginia, Tennessee, Texas, West Virginia, and District of Columbia; *West*: Alaska, Arizona, California, Colorado, Hawaii, Idaho, Montana, Nevada, New Mexico, Oregon, Utah, Washington, and Wyoming.

The age-adjusted cancer death rates increased significantly from 1970 to 1990 in each census region in the United States. The rate increased an average of 0.16% per year in the Northeast, 0.38% in the Midwest, 0.71% in the South, and 0.27% in the West. Since 1990, the rates have decreased at an ever faster rate, down on average by 1.41% in the Northeast, 1.02% in the Midwest, 1.15% in the South, and 1.30% in the West each year. At the beginning of the period, rates were highest in the Northeast, but since the late 1990s, rates in the South and Midwest have been higher. Throughout the period, the rates were lowest in the West census region.

**Source:** National Vital Statistics System. Mortality public use data files, 1970–2013. Available at http://www.cdc.gov/nchs/data_access/vitalstatsonline.htm.

**Reported by:** Jiaquan Xu, MD, jax4@cdc.gov, 301-458-4086.

